# Susceptibility of calf lung slice cultures to H5N1 influenza virus

**DOI:** 10.1080/22221751.2024.2432368

**Published:** 2024-12-03

**Authors:** Kate Guilfoyle, Monica Mirolo, Geert van Amerongen, Guido van der Net, Mara Sophie Lombardo, Theresa Störk, Guus Rimmelzwaan, Martin Ludlow, Albert Osterhaus

**Affiliations:** a Preclinical Speciality Services, Cerba Research (formerly Viroclinics Xplore), Schaijk, The Netherlands; b Research Center for Emerging Infection and zoonoses, University of Veterinary Medicine Hannover, Hannover, Germany

**Keywords:** Influenza, HPAI H5N1, lung slices, cattle, species susceptibility

## Abstract

The current outbreak of HPAI H5N1 virus infections in dairy cattle in the USA underscores the need for easily accessible methods to rapidly assess host susceptibility for infection with known and emerging influenza viruses. Here we show that *ex vivo* lung slice cultures from calves provide a useful method to rapidly screen host susceptibility to a range of influenza A viruses.

## Dear editor,

Recently highly pathogenic avian influenza (HPAI) A/H5N1 virus emerged as an important pathogen for dairy cattle in the USA. Movement of asymptomatic livestock has contributed to the ongoing outbreak which has currently spread among more than 280 dairy herds in 14 states [1]. The virus belongs to clade 2.3.4.4b, genotype B3.13, a reassortant between recent Eurasian H5N1 and American wild bird low pathogenic avian influenza (LPAI) lineages [2]. Clinical signs in infected cattle have been generally moderate thus far, although severe reduction in milk production and mortality have occurred. Virus transmission was assumed to occur via contaminated milking equipment rather than via the respiratory route, although recent experimental infections of calves have shown that the aerosol route of virus inoculation can result in a productive infection [3]. To date, six dairy farmers have been infected [4], illustrating the risk associated with exposure to unpasteurized milk or milk products. The risk of adaptation of bovine HPAIV A/H5N1 to mammals is considered low but the virus possesses features that may facilitate transmission among, and adaptation to mammals, including humans [5].

In the past four years, HPAI H5N1 viruses of multiple genotypes have caused numerous waves of unprecedented high-mortality outbreaks among birds and mammalian populations, while also necessitating mass cullings of poultry worldwide [1]. The unexpected appearance of a HPAI H5N1 virus in ruminants requires alternative strategies to more rapidly assess the susceptibility of different domestic mammalian species to infection with existing or newly emerging influenza A viruses.

Although animal infection experiments are the most informative route towards assessing this susceptibility, this requires animal infection facilities and permits, with adequate ethical, biosafety and biosecurity conditions. *In vitro* or *ex vivo* infection experiments, testing the permissibility of cell or organ cultures from virus target organs, can provide a first and important indication of species susceptibility. Immortalized cells and differentiated airway epithelial cells grown at air–liquid interface may provide valuable information [6]. Obviously, the former provide limited information, while the latter requires considerable preliminary preperatory work for multiple species.

Here, we present the use of lung slices obtained from calves after slaughtering, as readily available multi-cell organ cultures that may, without major adaptations, be a strategy that can also be used for other species.

Lung tissue was collected from two male 7- to 8-month-old calves (destined for human consumption) directly after slaughter for lung slice preparation, as described previously [7] and in the Appendix. Slices were infected with 10^5 TCID_50_/0.1 mL influenza virus by direct inoculation. Two HPAI H5N1 strains (A/Indonesia/5/2005 clade 2.1.3.2 and A/grey seal/Netherlands/302579/2023 clade 2.3.4.4b), a 2021–2022 Northern hemisphere A/H1N1 strain represented in the seasonal vaccine (A/Victoria/2570/2019), the A/H1N1pdm 2009 strain (A/Netherlands/602/2009) and a seasonal A/H3N2 strain (A/Netherlands/063/2011) were used. These viruses represent both group 1 and 2 subtypes, historical and current HPAI H5N1 virus isolates, as well as seasonal epidemic and pandemic strains. Following incubation with the inoculum for 90 min at 37°C, slices were rinsed and placed in fresh culture medium for incubation at 37°C. Slices were processed for virological and immune-histochemical analyses three days post-infection.

For virological analyses, slices were homogenized and clarified by low-speed centrifugation. Infectivity titres (log_10_ TCID_50_/g tissue) of supernatants were determined by titration on confluent MDCK cell monolayers, and read by agglutination of turkey red blood cells, as described previously [8]. Viral RNA levels (log_10_ copies(CP)/g) of supernatants were determined using previously designed primers [9]. Further information on the validation of the RT-qPCR assay is provided in the Appendix. Individual lung slice weight and sample volumes were used to calculate the infectivity and viral RNA titres per gram of tissue. Statistical analysis was performed using a Mann–Whitney U test.

**Figure 1.**
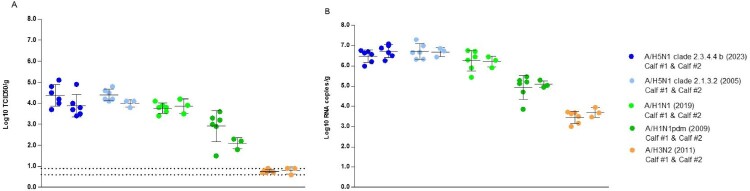
Viral load in calf lung slices infected with different influenza strains. (A) Individual infectivity titres (log_10_ TCID_50_/g) with group mean +/ – standard deviation (black lines). Lower limit of detection for each slice incorporates individual tissue weight and ranges between 0.6 and 0.9 log_10_ TCID_50_/g, indicated by dashed lines. (B) Individual viral RNA levels (log_10_ CP/g) with group mean +/ – standard deviation (black lines) shown. *N* = 6 per calf for A/H5N1 clade 2.3.4.4b (2023) infection; *n* = 6 calf #1, *n* = 3 calf #2 for all other infections. Statistical analysis is provided in Supplementary Appendix, Figure S1.

The results of the infectivity titrations and the RT-qPCR analysis are shown in Figure 1. Slices from both donor calves were permissive for replication of A/H5N1 clade 2.3.4.4b (2023), A/H5N1 clade 2.1.3.2 (2005), A/H1N1 (2019) and A/H1N1pdm (2009) influenza viruses. No replication competent virus was detected in lung slices infected with the A/H3N2 (2011) influenza virus (Figure 1A). Viral RNA was detected in all samples (Figure 1B). Lung slices from donor calf #2 (*n* = 3) were mock infected with culture medium, there were no detectable titres (log10 TCID50/g or log10 CP/g) in these slices (data not shown).

Results correlated well between infectious titres and RNA levels (Appendix, Figure S2A). Overall, there was no statistical difference between infectivity or viral RNA titres of lung slices infected with either the A/H5N1 strain or A/H1N1 (2019) influenza viruses. Significant differences were observed in the levels of replication competent virus and viral RNA detected when infected with either the A/H3N2 (2011) or A/H1N1pdm (2009) strains, in comparison to those infected with the other influenza virus strains (Appendix, Figure S2B and S2C).

The preservation status of the slices was not optimal for IHC staining, resulting in loss of tissue, but limited influenza nucleoprotein antigen detection showed virus replication in epithelial cells of bronchi and bronchioles. Appendix, Figure S2.

## Discussion

The ongoing outbreak of HPAI H5N1 virus clade 2.3.4.4b infection in dairy cattle, as well as HPAI H5N1 virus detection in various other mammalian species including humans, underscore the need for effective surveillance systems and suitable methods to rapidly assess host susceptibility to infection with various known and emerging influenza viruses. This understanding is critical for assessing the risk these viruses may pose to animal and human health, including their panzootic and pandemic potential.

Our findings demonstrate that lung slices are an accessible source material for implementing an effective screening tool to rapidly assess the replication competence of existing and emerging influenza A viruses of bovine lower respiratory tract tissue. This ability to compare AIV strains in this *ex vivo* model provides a more rapid alternative to the recently reported AIV infection of primary bovine bronchial epithelial cells cultured on air–liquid interface [10]. This *ex vivo* method proved to be sensitive in detecting the replication potential of a range of influenza A viruses in lung tissue, with minimal variation between slices and between donor animals (Figure 1).

In summary, this fast, accessible lung slice culture method provides information relevant to the potential replication of Influenza A subtypes in the bovine species. It may be used to test the susceptibility of lungs and possibly other organs of many animal species and for a range of influenza A viruses of avian or mammalian origin. We have also successfully used lung slice cultures of ferrets to assess replication competence for a range of influenza viruses (data not shown). It would be of further interest to use *ex vivo* lung and possibly mammary gland slice cultures [11] of different other animal species to rapidly screen their permissiveness for replication of influenza viruses.

## Supplementary Material

EMI_Suceptibility of calf lung slice cultures to H5N1 infection_Supplementary Appendix_Revised 14 Nov 2024.docx
