# Supplementary material for: Susceptibility of calf lung slice cultures to H5N1 influenza virus
Source: Emerg Microbes Infect. 2024 Dec 3;14(1):2432368. doi: 10.1080/22221751.2024.2432368 (PMC11632935; doi:10.1080/22221751.2024.2432368)
Supplement: EMI_Suceptibility of calf lung slice cultures to H5N1 infection_Supplementary Appendix_Revised 14 Nov 2024.docx [file TEMI_A_2432368_SM8124.docx]

**Supplementary Appendix**

Supplementary Figures 1

Figure S1. Statistical analysis. 1

Figure S2. Detection of influenza A virus (AIV) in ex vivo lung slice cultures 2

Supplementary Methods 3

Lung slice preparation 3

Validation of an RT-qPCR assay for AIV 4

**Supplementary Figures**


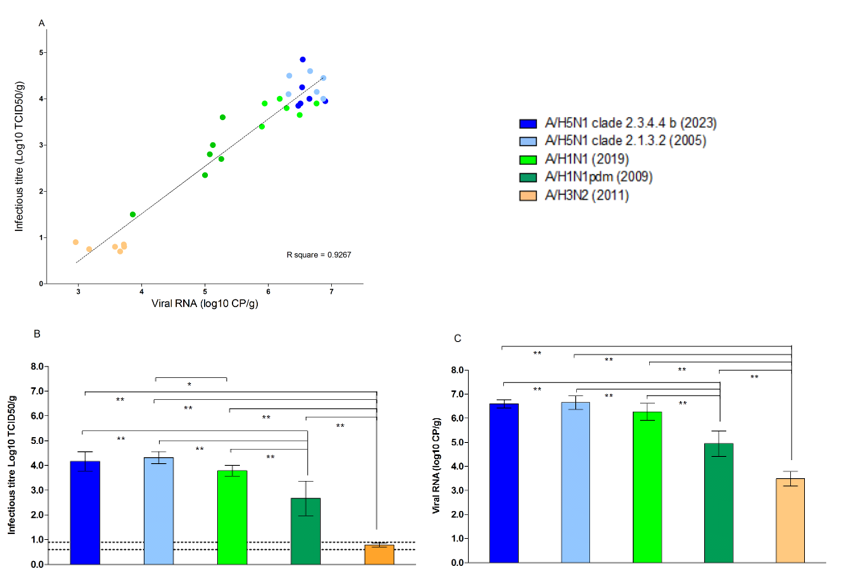


**Figure S1.** Statistical analysis. Viral load of lung slices three days post infection with different influenza viruses, titres from calf #1 and calf #2, shown separately in Figure 1, were averaged. (A) linear regression analysis performed to show correlation between infectivity (log_10_ TCID_50_/g) and viral RNA (log_10_ CP/g) levels. (B) Mean infectivity titres (log_10_ TCID_50_/g), group mean +/- standard deviation (black lines) shown with statistically significant differences indicated (*P* < .01 **; *P* < .05 *). (C) Mean viral RNA titres (log_10_ CP/g), group mean +/- standard deviation (black lines) shown with statistically significant differences indicated (*P* < .01 **; *P* < .05 *).


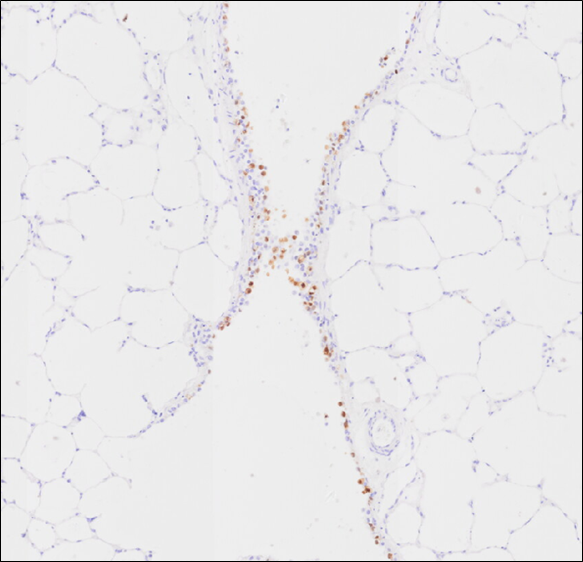


**Figure S2.** Detection of influenza A virus (AIV) in ex vivo lung slice cultures. Immunohistochemical staining detecting AIV nucleoprotein antigen (mouse monoclonal antibody CLONEGENE LLC, HB65) within epithelial cells of bronchi and bronchioli of a calf lung slice (donor calf #2) infected with HPAI A/H5N1 (clade 2.1.3.2) 2005 virus.

**Supplementary methods**

**Lung slice preparation**

Lung tissue was collected at the time of slaughter of two 7- to 8-month-old healthy calves fit for human consumption. No specific pre-screening for previous exposure to influenza virus or other respiratory pathogens was performed as lung tissue appeared normal with no obvious macroscopic pathology or signs of disease. Lung tissue was injected with medium-agarose mix, composed 1:1 (vol/vol) of DMEM-F12 medium (ATCC cat. #30-2006) with 10% FBS (Cat#: FBS-12A), 1% Penicillin/streptomycin (Sigma; Cat#: P4333), 1% Amphotericin B (Sigma; Cat#: A2942) warmed to 40^o^C and 4% molten agarose (Sigma; Cat.#: A4718) in PBS (Sigma; Cat#: D8537), cooled to 40^o^C. Lung tissue was placed in PBS and cooled on wet ice for approximately four hours. Lung slices were prepared manually, ensuring that both bronchiolar and alveolar cells are present in each slice. They were placed in 6-well tissue culture plates, 3.0 mL of medium (described above) added and incubated at 37^o^C overnight. Approximately 24 hours after preparation, media was removed, slices were rinsed with 1.5mL fresh medium, placed in fresh 6-well tissue culture plates and dried with sterile absorbent tissue. Lung slices were subsequently infected as described in main text. For infection with the A/H5N1 clade 2.3.4.4b (2023) strain, n = 6 slices from both donor calf #1 and donor calf #2. For infection with all other virus strains, n = 6 slices from donor calf #1 and n = 3 slices from donor calf #2. Following a 90-minute incubation period at 37^o^C, slices were rinsed with 1.5 mL of medium, placed in fresh plates and 3.0 mL culture medium added prior to incubation at 37^o^C.

**Validation of an RT-qPCR assay for AIV**

The number of RNA copies of each influenza A virus strain was calculated using a positive control standard curve which was generated during validation of the RT-qPCR assay. Positive-control RNA was generated via in vitro transcription of a cloned 95-bp fragment of influenza A virus, which had been inserted downstream the T7 promoter in a pcDNA3.1+ mammalian expression vector. Briefly, the positive dsDNA control was linearized by XhoI restriction enzyme overnight at 37°C. The DNA (1μg) was in vitro transcribed using RNA the HiScribe T7 high-yield RNA synthesis kit (NEB) according to manufacturer’s instructions. The RNA yield was determined photometrically from 2 μL of RNA at an absorbance of 260 nm and 280 nm with a Thermo Scientific™ Multiskan™ GO Microplate Spectrophotometer. The copy number of the newly generated RNA was determined using the equation (amount RNA (ng/µl) x 6.022 x 10^23/ (RNA fragment length (bp) x 109 x 340) = Y (RNA copies/µl). The RNA that was generated from positive control was tested in the RT-qPCR assay using published primers [8]. All RT-qPCRs were performed using the Luna universal probe one-step RT-qPCR kit (NEB) using a concentration of 0.4 μM for each primer and 0.2 μM for the probe in the RT-qPCR master mix. Assays were performed using a LightCycler 96 Real-Time PCR System (Roche) with an initial 10-min RT step at 55°C, followed by incubation for 1 min at 95°C. Forty-five two-step cycles were then performed using the following conditions: denaturation at 95°C for 10 s, annealing and amplification at 60°C for 30 s. The fluorescence level was detected and quantified. To produce the standard curves, the RNA from the positive control was diluted 1000-fold to reach a concentration of 1.73 x 10^10^ RNA copies/μl, and serially 10-fold diluted (10^-1^ to 10^-12^). Duplicated of each dilution were tested in the RT-qPCR assay alongside RNA extracted from influenza A infected calf lung slices from donor 1 and donor 2 in two separate RT-qPCR assays. The standard curves were automatically produced in the LightCycler 96 Software (Roche). The standard curves for the positive control showed a slope of -3.223 and -3.232 for donor 1 and donor 2 respectively. For both curves the R^2 value was 1.
